# Whole-Genome Linkage Scan Combined With Exome Sequencing Identifies Novel Candidate Genes for Carotid Intima-Media Thickness

**DOI:** 10.3389/fgene.2018.00420

**Published:** 2018-10-09

**Authors:** Dina Vojinovic, Maryam Kavousi, Mohsen Ghanbari, Rutger W. W. Brouwer, Jeroen G. J. van Rooij, Mirjam C. G. N. van den Hout, Robert Kraaij, Wilfred F. J. van Ijcken, Andre G. Uitterlinden, Cornelia M. van Duijn, Najaf Amin

**Affiliations:** ^1^Department of Epidemiology, Erasmus MC University Medical Center, Rotterdam, Netherlands; ^2^Department of Genetics, School of Medicine, Mashhad University of Medical Sciences, Mashhad, Iran; ^3^Department of Cell Biology, Center for Biomics, Erasmus MC University Medical Center, Rotterdam, Netherlands; ^4^Department of Internal Medicine, Erasmus MC University Medical Center, Rotterdam, Netherlands; ^5^Nuffield Department of Population Health, University of Oxford, Oxford, United Kingdom

**Keywords:** atherosclerosis, intima-media thickness, genetics, linkage, exome sequencing

## Abstract

Carotid intima-media thickness (cIMT) is an established heritable marker for subclinical atherosclerosis. In this study, we aim to identify rare variants with large effects driving differences in cIMT by performing genome-wide linkage analysis of individuals in the extremes of cIMT trait distribution (>90th percentile) in a large family-based study from a genetically isolated population in the Netherlands. Linked regions were subsequently explored by fine-mapping using exome sequencing. We observed significant evidence of linkage on chromosomes 2p16.3 [rs1017418, heterogeneity LOD (HLOD) = 3.35], 19q13.43 (rs3499, HLOD = 9.09), 20p13 (rs1434789, HLOD = 4.10), and 21q22.12 (rs2834949, HLOD = 3.59). Fine-mapping using exome sequencing data identified a non-coding variant (rs62165235) in *PNPT1* gene under the linkage peak at chromosome 2 that is likely to have a regulatory function. The variant was associated with quantitative cIMT in the family-based study population (effect = 0.27, *p*-value = 0.013). Furthermore, we identified several genes under the linkage peak at chromosome 21 highly expressed in tissues relevant for atherosclerosis. To conclude, our linkage analysis identified four genomic regions significantly linked to cIMT. Further analyses are needed to demonstrate involvement of identified candidate genes in development of atherosclerosis.

## Introduction

Cardiovascular diseases, including heart and cerebrovascular diseases, are listed among the leading causes of death in developed countries (Xu et al., 2016). The underlying pathology in the majority of cases is atherosclerosis (Falk, 2006). cIMT, a quantitative measure of carotid artery wall thickening, is a marker for subclinical atherosclerosis that has been shown to predict future cardiovascular events in large epidemiological studies (Lorenz et al., 2007; Polak et al., 2011; Den Ruijter et al., 2012). cIMT is determined by both traditional cardiovascular risk factors, such as aging, blood pressure, BMI, plasma lipid levels, diabetes mellitus or smoking, and genetic factors (Lusis, 2012). Genetic factors play a key role in the etiology of cIMT with heritability estimates ranging from 30–60% (Fox et al., 2003; Sacco et al., 2009). Several genome-wide linkage studies of quantitative cIMT published up to date reported significant and suggestive evidence of linkage on chromosomes 2q33-q35, 6p12-p22, 7p, 11q23, 12q24, 13q32-q33, and 14q31 (Fox et al., 2004; Wang et al., 2005; Sacco et al., 2009; Kuipers et al., 2013). The largest genome-wide association study (GWAS) of cIMT, including 42,484 individuals, identified only three genomic regions of common non-coding genetic variation on 8q24 (near *ZHX2*), 19q13 (near *APOC1*), and 8q23.1 (*PINX1*) and an additional suggestive region on 6p22 (near *SLC17A4*; Bis et al., 2011). In addition, an exome-wide association study in 52,869 individuals identified the association of protein-coding variants in *APOE* with cIMT (Natarajan et al., 2016). The identified variants provide valuable insights into the genetic architecture of cIMT but explain a small proportion of the trait variance (Bis et al., 2011). A previous sequencing study of cIMT candidate regions in population-based cohorts yielded inconclusive results due to limited power (Bis et al., 2014). A more powerful approach for uncovering the role of rare variants is a family-based study design due to the higher frequency of the rare variants (Auer and Lettre, 2015). The chances of success for family-based studies are even higher in genetic isolates since rare variants become more frequent due to founder effect, genetic drift, and inbreeding (Stacey et al., 2011; Gudmundsson et al., 2012; Auer and Lettre, 2015).

In this study, we hypothesized that there may be rare variants with large effects driving differences in cIMT independently of traditional cardiovascular risk factors and that these variants are enriched in the extremes of the cIMT distribution. To the best of our knowledge, no study to date explored extremes of quantitative cIMT. However, this approach has been demonstrated as successful for some other quantitative traits. Following the same approach as described in our study, Amin et al. (2018) successfully identified a rare variant of large effect in large extended families. To discover such variants in the extremes of cIMT distribution, we performed affected-only genome-wide linkage analysis of cIMT followed by fine-mapping using exome sequencing in a large family-based study from a genetically isolated population in the Netherlands.

## Materials and Methods

### Study Population

Our study population consisted of participants from ERF study. ERF is a family-based cohort that includes around 3,000 inhabitants of a genetically isolated community in the South-West of Netherlands (Pardo et al., 2005). The community was constituted as a religious isolate at the middle of the 18th century by a limited number of founders (Pardo et al., 2005). The population has remained in isolation with minimal immigration rate and high inbreeding (Aulchenko et al., 2004; Pardo et al., 2005). All ERF participants are living descendants of a limited number of founders living in the 19th century. The Medical Ethical Committee of the Erasmus University Medical Center, Rotterdam, approved the study. Written informed consent was obtained from all participants.

### Phenotypes

Participants from ERF underwent extensive clinical examination between 2002 and 2005. cIMT was measured using high-resolution B-mode ultrasonography with a 7.5-MHz linear array transducer (ATL UltraMark IV). Maximum cIMT was measured on the three still, longitudinal, two-dimensional ultrasound images of the near and far wall from both left and right arteries, as described previously (Sayed-Tabatabaei et al., 2005). The mean value of these measurements was used for the analyses.

Information on covariates included age, sex, and smoking status. BMI was defined as weight divided by the square of height (kg/m^2^) and WHR was computed by dividing the waist and hip circumferences with each other. Hypertension was defined as systolic blood pressure above 140 mmHg, diastolic blood pressure above 90 mmHg, or use of medication for treatment of hypertension. Dyslipidemia was defined as total cholesterol above 6.2 mmol/L or use of lipid-lowering medication, whereas diabetes was defined as fasting plasma glucose levels above 7 mmol/L, random plasma glucose above 11.1 mmol/L, or use of medication indicated for treatment of diabetes.

### Genotyping

#### Genotyping on the Illumina 6K Array

Genomic DNA was extracted from peripheral venous blood of all study participants using the salting out procedure (Miller et al., 1988). Genotyping was performed using the 6K Illumina Linkage IV Panels (Illumina, San Diego, CA, United States) at the Centre National de Genotypage in France. Markers with a MAF < 5%, call rate < 98%, or which failed an exact test of HWE (*p*-value < 10^-8^) were removed during the quality control process. In total, 5,250 autosomal variants were available for analysis.

#### Exome Sequencing

The exomes of randomly selected participants from the ERF study were sequenced at the Cell Biology Department of the Erasmus University Medical Center, Rotterdam. Sequencing was performed at a median depth of 57× using the Agilent version V4 capture kit on an Illumina Hiseq2000 sequencer using the TruSeq Version 3 protocol (Amin et al., 2016, 2017a). After quality control, we retrieved 528,617 SNVs in 1,308 individuals, of which 1,046 had cIMT data available. Annotation of the SNVs was performed using the SeattleSeq annotation database^1^. To further assess the functionality of the variants, we used RegulomeDB database that annotates SNVs with known and predicted regulatory elements and CADD tool for scoring the deleteriousness of variants (Boyle et al., 2012; Kircher et al., 2014). The ERF data are available in the European Genome-phenome Archive (EGA) public repository with ID number EGAS00001001134.

### Statistical Analysis

#### Linkage Analysis

We performed affected only genome-wide multipoint NPL analysis in MERLIN 1.1.2 using individuals from the ERF study (Abecasis et al., 2002). Individuals that scored above the 90th percentile of the distribution of the residuals from the regression of cIMT onto age, age^2^, sex, smoking status, BMI, WHR, diabetes, dyslipidemia, and hypertension were set as affected (*N* = 103). Descriptive characteristics of the selected individuals are presented in **Table 1**. The selected individuals were older and higher cIMT measurements compared to all ERF study participants (**Table 1**). They also had a higher prevalence of hypertension, dyslipidemia, and diabetes than all ERF study participants, whereas the BMI and WHR were comparable (**Table 1**). These 103 affected individuals were connected to each other in a large pedigree consisting of 5,083 individuals. To facilitate linkage analysis, the 103 affected individuals were clustered into 21 smaller non-overlapping sub-pedigrees with a maximum bit size of 24 using the PEDCUT software version 1.19 (Liu et al., 2008). Bit size value is used to characterize the maximal number of subjects of interest who share a common ancestor (Liu et al., 2008). The number of affected subjects of interest in the sub-pedigrees ranged from two to eight. MEGA2 software tool version 4.4 (Baron et al., 2014) was used to create input files for MERLIN. Mendelian inconsistencies were set to missing within the whole sub-pedigree. There were 543 Mendelian inconsistencies observed among 5,250 autosomal variants. After they were set to missing, 4,707 autosomal variants were used in the linkage analysis. We also performed affected only parametric linkage analysis under the dominant and recessive models assuming incomplete penetrance of 0.5 and a disease allele frequency of 0.01 using MERLIN. Marker allele frequencies were calculated from all genotyped individuals in the pedigrees. Subsequently, we carried out per family analyses in order to identify families that were contributing predominantly to the linkage signals, henceforth referred to as “contributing families.” Additionally, we performed variance component linkage analysis in MERLIN using quantitative cIMT in the total study population. To facilitate analysis, PEDCUT software was used to cluster individuals into 116 non-overlapping sub-pedigrees. The number of subjects of interest in the sub-pedigrees ranged from 2 to 18. To determine the significance of each test, the LOD score was calculated as the log10 of the likelihood ratio. The LOD score of 3.3 or higher was considered to represent genome-wide significance threshold, whereas the LOD score of 1.9 was used to declare genome-wide suggestive threshold (Ott et al., 2015).

**Table 1:** Descriptive statistics of study populations including ERF cases (*N* = 103) selected for the linkage analysis and ERF overall.

Characteristics	ERF cases	ERF overall
Age, mean *(SD)*	53.6 (13.6)	48.3 (14.2)
Gender, % of males	45.6%	40.2%
cIMT (mm), mean *(SD)*	1.1 (0.2)	0.8 (0.2)
Smoking, % of ever smokers	44.7%	41.9%
BMI (kg/m^2^), mean *(SD)*	26.9 (3.7)	26.7 (4.4)
WHR, mean *(SD)*	0.9 (0.1)	0.9 (0.1)
Hypertension, % of cases with hypertension	63.1%	48.7%
Dyslipidemia, % of cases with dyslipidemia	51.5%	36.2%
Diabetes, % of patients with diabetes	6.8%	4.5%

*cIMT, carotid intima-media thickness; BMI, body mass index; WHR, waist to hip ratio; hypertension, systolic blood pressure above 140 mmHg, diastolic blood pressure above 90 mmHg, or use of medication for treatment of hypertension; dyslipidemia, total cholesterol above 6.2 mmol/L, or use of lipid-lowering medication; diabetes, fasting plasma glucose levels above 7 mmol/L, random plasma glucose above 11.1 mmol/L, or use of medication indicated for the treatment of diabetes*.

#### Identification of Variants Under the Linkage Peaks Using Exome Sequencing

We used exome sequence data to identify variants that could explain observed linkage peaks. To this end, we looked for variants that were shared among the majority of affected individuals from the contributing families within the respective linkage peak. We only considered variants with MAF < 5% or absent in 1kG and MAF < 5% in the ERF controls which were defined as individuals who scored below the mean of the distribution of the residuals from the regression of cIMT onto age, age^2^, sex, smoking status, BMI, WHR, diabetes, dyslipidemia, and hypertension. The MAF of variants absent in 1kG project was checked in NHLBI Exome Sequencing Project^2^. Candidate variants were subjected to quantitative trait association analysis with cIMT in the ERF under the same model as in the sharing analysis (additive, dominant, recessive) using the RVtests software (Zhan et al., 2016). Inverse normalized residuals from the regression of cIMT onto age, age^2^, sex, smoking status, BMI, WHR, diabetes, dyslipidemia, and hypertension were used in the association analysis. To take into account multiple tests, we first calculated a number of independent tests using the method of Li and Ji (2005). Subsequently, Bonferroni corrected *p*-value was calculated based on number of independent tests. GTEx portal^3^ was used to check for gene expression.

## Results

**FIGURE 1:**
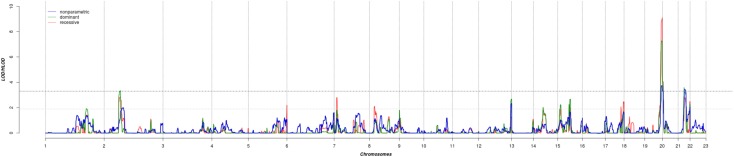
The results of genome-wide linkage scan for non-parametric (blue) and parametric analyses under the dominant (green) and recessive (red) models. The *x*-axis shows 22 autosomal chromosomes, whereas the *y*-axis shows the LOD scores for non-parametric model and heterogeneity LOD (HLOD) scores for dominant and recessive model. Black dotted line depicts the genome-wide significant threshold, whereas gray dotted line shows the suggestive threshold.

The results of affected only genome-wide NPL and parametric linkage scans are illustrated in **Figure 1**. Regions with significant (LOD > 3.3) evidence of linkage in either the non-parametric or the parametric analyses are shown in **Table 2**. Significant evidence of linkage for cIMT was observed to chromosomes 2p16.3, 19q13.43, 20p13, and 21q22.12 in the parametric linkage analysis under the dominant model, and to chromosome 19q13.43 and 20p13 in the parametric linkage analysis under the recessive model. The families contributing predominantly to these linkage peaks and the distribution of their per-family HLOD scores are shown in **Supplementary Figures S1–S4**.

**Table 2:** Genome-wide significant results of linkage analyses for cIMT.

Region	Start SNV	End SNV	Start position^∗^	End position^∗^	SNV with max LOD	Dominant (HLOD)	Recessive (HLOD)	Non-parametric (LOD)
2p16.3	rs1447107	rs1017267	45272197	56785785	rs1017418	**3.35**	2.88	1.40
19q13.43	rs897783	rs3499	52031162	59093484	rs3499	**7.17**	**9.09**	**3.73**
20p13	rs1434789	rs241605	137900	3915064	rs1434789	**4.10**	**3.87**	**3.34**
21q22.12	rs762173	rs2836803	33832675	40351780	rs2834949	**3.59**	1.86	2.14

*SNV, single nucleotide variant; HLOD, heterogeneity LOD. Regions with significant evidence of linkage are in bold. ^∗^Start and end positions correspond to genetic position of SNV at the start or end of the base to base linkage region according to hg19 assembly. Start and end SNVs are reported for base to base linkage regions*.

We next determined to what extent the affected members in these families shared rare variants under the linkage peaks. Sharing analyses under the base to base linkage peak at chromosome 2 (family specific HLOD = 3.63) identified intronic and coding-synonymous variants (**Table 3**). The most interesting finding is a variant (rs62165235) with MAF 0.038 in 1kG mapping to *PNPT1*. The variant, shared by six out of eight affected relatives, is likely having a regulatory function and affecting transcription factor binding and matched DNase Footprinting and DNase sensitivity (Category 2b Regulome DB score; **Tables 3**, **4**). The variant was sequenced at a read depth of 37x and it showed significant association with quantitative cIMT in the ERF (effect = 0.27, *p*-value = 0.013, **Table 3**) after applying Bonferroni correction (*p*-value = 0.05/3 independent tests = 0.017). The effect estimate of the minor allele C on untransformed cIMT suggested a mean increase of 0.04 mm for each minor allele (0.04 mm for heterozygote C/T carriers and 0.08 mm for homozygous C/C carriers; **Table 3**). This variant explained 0.3% of variation in the ERF.

**Table 3:** Variants shared among the affected family members of the family that predominantly contributed to the LOD score at chromosome 2.

Name^∗^	Function	Gene	MAF ERF controls^∗∗^	MAF 1kG^∗∗∗^	CADD	Regulome DB	Effect allele	Association analysis in ERF			
								Beta	Beta_untransformed_	*SE*	*P*
rs375801385	Intron	*FBXO11*	0.031	0.056	17.18	6	T	0.023	0.014	0.145	0.876
2:48848294	Intron	*GTF2A1L*	0.031	NA	5.82	–	C	0.023	0.014	0.145	0.876
rs149304214	Coding-synonymous	*SPTBN1*	0.016	0.002	15.95	–	T	0.311	0.059	0.182	0.087
rs62165235	Intron	*PNPT1*	0.044	0.038	4.41	2b	C	0.265	0.037	0.107	0.013
rs114706375	Intron	*USP34*	0.026	0.010	0.12	5	C	0.124	0.036	0.145	0.393
rs144629927	Intron	*XPO1*	0.021	0.003	4.60	3a	G	0.131	0.041	0.159	0.409

*MAF, minor allele frequency; 1kG, 1000 Genomes; CADD, Combined Annotation-Dependent Depletion score; Regulome DB, Regulome DB score; beta, effect estimate; beta_*untransformed*_, effect estimate from association analysis in which untransformed cIMT was used; SE, standard error of beta; P, *p*-value; ERF, Erasmus Rucphen Family study. ^∗^The variants are ordered based on their genomic position (hg19 assembly). First four variants are shared by 6 of 8 affected family members in a family contributing predominately to the linkage peak, and last two variants are shared by five of eight affected family members. ^∗∗^ERF controls were defined as individuals who scored below the mean of the distribution of the residuals from the regression of cIMT onto age, age^*2*^, sex, smoking status, BMI, WHR, diabetes, dyslipidemia, and hypertension. ^∗∗∗^If MAF was unknown in 1kG, MAF reported from Exome Sequencing Project if available*.

**Table 4:** Functional annotation of rs62165235 variant and variants that are in LD (*r*^2^ > 0.6) using HaploReg 4.1 (Ward and Kellis, 2012).

Variant	LD (r^2^)	Ref	Alt	GERP cons^∗^	Promoter histone marks	Enhancer histone marks	DNAse	Proteins bound	Motifs changed	Selected eQTL hits	RefSeq genes	Function
rs7591128	0.65	T	G	No	–	BLD	–	–	Six altered motifs	One hit	39 kb 5^′^ of *CCDC88A*	Intergenic
rs78928997	0.75	T	A	Yes	–	–	–	–	Seven altered motifs	–	*SMEK2*	3^′^-UTR
rs62165193	0.77	C	T	No	–	Two tissues	–	–	GR	–	*SMEK2*	Intronic
rs62165227	0.61	G	A	No	–	–	–	–	–	–	*PNPT1*	Intronic
rs62165231	0.95	C	T	No	–	–	HRT	–	Rad21,Tgif1	One hit	*PNPT1*	Intronic
rs62165235	1	T	C	No	24 tissues	–	15 tissues	E2F6	Seven altered motifs	–	*PNPT1*	Intronic
rs62165236	0.96	T	C	No	–	–	–	–	ZID	–	2.1 kb 5^′^ of *PNPT1*	Intergenic
rs79873145	0.76	T	C	No	–	GI	–	–	Four altered motifs	One hit	30 kb 5^′^ of *PNPT1*	Intergenic

*Ref, reference allele; Alt, alternative allele; GERP, conservation score. ^∗^GERP conservation score indicates whether the element is conserved or not according to the algorithm. The variant highlighted in red was identified in the sharing and association analysis in the ERF*.

**FIGURE 2:**
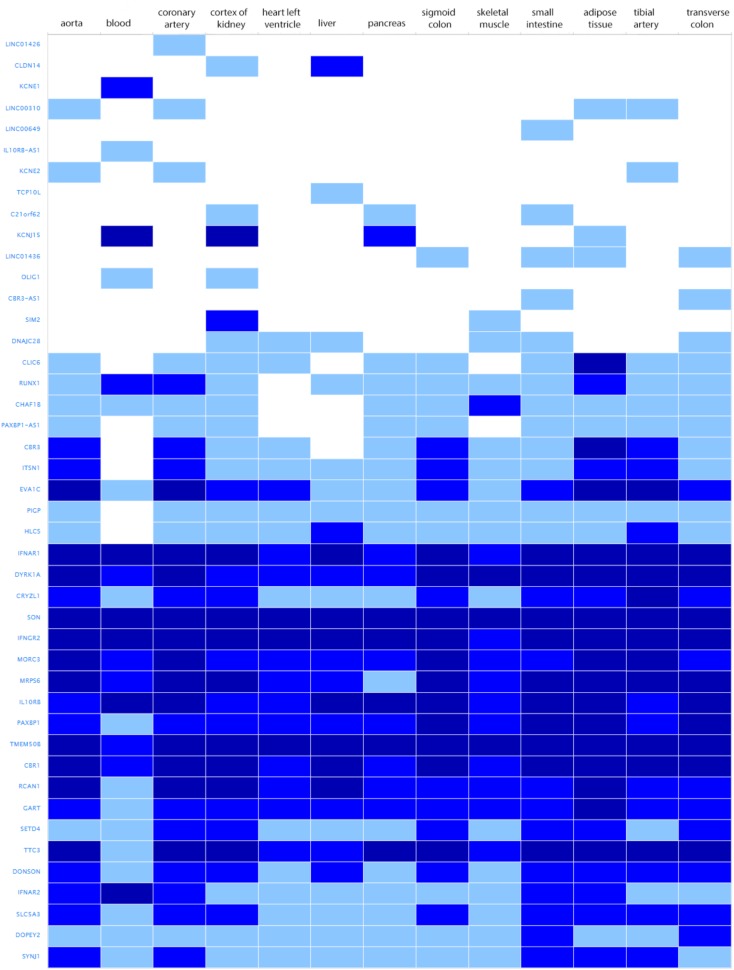
Genes under the base to base linkage peak at chromosome 21 and their expression levels in tissues relevant for atherosclerosis according to GTEx database. Gene names are shown on *y*-axis and tissues on *x*-axis. Color depicts expression level estimated as fragments per kilobase of exon model per million reads mapped (FPKM). The gray box stands for expression level below cutoff (0.5 FPKM), light blue box stands for low expression level (between 0.5 and 10), medium blue for medium expression level (between 11 and 1000 FPKM), dark blue for high expression level (more than 1000 FPKM or more than 1000 TPM), and white for no data available.

The search for shared variants within the linkage regions at chromosome 19, 20, and 21 identified several variants to be shared among the affected family members; however, none of the variants showed significant association with quantitative cIMT (**Supplementary Tables S1–S3**). There are, however, several potentially interesting candidate genes for atherosclerosis in each of these linked regions, for instance, among the genes under the base to base linkage peaks at chromosome 19 and 20, several genes have been implicated in the pathogenesis of cardiovascular disease, including *FCAR, TNNT1, OSCAR, FPR2* under the peak at chromosome 19 and *ADAM33, TRIB3, HSPA12B* under the peak at chromosome 20. The linkage peak at chromosome 21 harbors several genes that are highly expressed in tissues relevant for atherosclerosis (e.g., vascular, liver, kidney, gastrointestinal, and adipose tissue; Ramsey et al., 2010), including *IFNAR1, DYRK1A, SON, IFNGR2, MORC3, MRPS6, IL10RB, TMEM50B, CBR1, RCAN1*, and *TTC3* (**Figure 2**). According to the Ingenuity Pathway Analysis (IPA) tool (QIAGEN Inc.^4^), which exposes possible functional relationship between the genes by expanding upstream analysis to include regulators that are not directly connected to targets in the dataset, these genes connected to a network illustrated in **Supplementary Figure S5**.

There were several regions that showed suggestive evidence of linkage, including 1q31.1, 5q35.3, 7p21.3, 8p22, 12q24.33, 14q22.2, 15q21.3, and 17q25.3. As the region at chromosome 12 has previously been linked to cIMT, we have explored it further (**Figure 1**). Search for shared variants within this region identified no variants that can explain linkage signal.

The results of linkage analysis when using cIMT as a quantitative outcome are shown in **Supplementary Figure S6**.

## Discussion

In this study, we have identified genomic regions at 2p16.3, 19q13.43, 20p13, and 21q22.12 with significant evidence of linkage to cIMT. These regions have not been reported before. Identification of variants under the linkage peaks using exome sequencing revealed a variant with likely regulatory function mapping to *PNPT1* gene at chromosome 2 and several candidate genes at chromosome 21.

As the present study targets genes with relatively large effects, we studied the extremes of cIMT distribution in the discovery population. Even though extreme trait approach neglects much of the overall distribution of the trait and some rare variants with the moderate effects may be missed, it has been shown that the power to detect rare variants can be increased due to an excess of rare variants in the upper tails of the distribution (Coassin et al., 2010; Lamina, 2011; Auer and Lettre, 2015). Comparison of the results obtained in the linkage analysis of the extremes of cIMT distribution and those using cIMT as a quantitative trait revealed no overlap, highlighting the power of the approach we have followed.

When comparing the results of several genome-wide linkage studies of cIMT that have been conducted so far, we noticed that a region with suggestive evidence of linkage in our study at chromosome 12 has previously been linked to cIMT through the linkage scan (Fox et al., 2004). Similarly to the previous study which identified 12q24, we did not identify variants by sharing analysis that could explain the linkage signal. Even though linkage findings from prior studies already showed limited generalizability across the reported linkage peaks due to selected nature of the cohorts, the overlap of our finding with the literature suggests that our study population is representative of the general population. However, the linkage signal at chromosome 12 is relatively weak in our population.

Among the regions with significant evidence of linkage to cIMT in our study, we identified 2p16.3 which gave significant linkage signal under the dominant model and suggestive signal under the recessive model. This region has previously been associated with PCOS and POAG (Liu et al., 2010; Mutharasan et al., 2013). Interestingly, women with PCOS are at a greater risk of premature atherosclerosis (Meyer et al., 2012), whereas atherosclerosis is associated with vascular conditions that are correlated with POAG (Belzunce and Casellas, 2004). The region has further been implicated in BMI (Akiyama et al., 2017) and glycated hemoglobin levels (Wheeler et al., 2017). Both obesity and poor glycemic control are risk factors for variety of diseases including atherosclerosis and cardiovascular diseases.

Identification of variants under the base to base peak at chromosome 2 using exome sequence data revealed a variant that lies in DNase sites, promotes histone marks and protein binding regions, and changes regulatory motifs based on the variant allele change. The variant is mapped to intron 1 of polyribonucleotide nucleotidyltransferase 1 (*PNPT1*) gene which encodes a protein predominantly localized in the mitochondrial intermembrane space and is involved in import of RNA to mitochondria^5^. *PNPT1* has been characterized as a type I interferon-inducible early response gene (Leszczyniecka et al., 2002, 2003). Type I interferons promote atherosclerosis by enhancing macrophage-endothelial cell adhesion and promoting leukocyte attraction to atherosclerosis-prone sites in animal models (Goossens et al., 2010). Even though our finding supports a role of *PNPT1* as a candidate gene in atherosclerosis, we acknowledge that *PNPT1* variant is unlikely to be causal and cannot explain the linkage signal at chromosome 2 to cIMT. Furthermore, we attempted to replicate the association of this variant with cIMT in the Rotterdam Study, a population-based cohort study (detailed information is provided in **Supplementary Material** and **Supplementary Table S4**). However, the variant was not available in the exome sequencing data of the Rotterdam Study.

The other interesting region includes the linkage peak at chromosome 21 which is also known as a Down critical region. Interestingly, persons with Down syndrome are protected against atherosclerosis, in spite of increases in metabolic disturbances and obesity in Down syndrome (Colvin and Yeager, 2017). Even though identification of variants using exome sequencing did not identify a causal variant, this region contains several plausible candidate genes which are highly expressed in relevant tissues (Ramsey et al., 2010), including *IFNAR1, DYRK1A, SON, IFNGR2, MORC3, MRPS6, IL10RB, TMEM50B, CBR1, RCAN1*, and *TTC3*. *DYRK1A* signaling pathway is linked to homocysteine cycle which is associated with an increased risk of atherosclerosis (Noll et al., 2009; Tlili et al., 2013). *IFNGR2* and *RCAN1* also play a role in atherosclerosis (Mendez-Barbero et al., 2013; Voloshyna et al., 2014). Notably, IPA analysis revealed that those genes are connected in one network, and directly or indirectly linked to *TP53*. *TP53* encodes a tumor suppressor gene p53 involved in regulation of cell proliferation and apoptosis. Numerous studies implicated p53 in development of atherosclerosis and vascular smooth muscle cell apoptosis (Tabas, 2001; Mayr et al., 2002; Mercer et al., 2005; Boesten et al., 2009; Varela et al., 2015). Higher plasma p53 levels were also associated with an increased cIMT (Chen et al., 2009). However, it is important to note that network analysis is based on the knowledge databases that are always evolving and new discoveries happen all time.

Furthermore, we identified 19q13.43 and 20p13 regions with significant evidence of linkage to cIMT. Several genes under the linkage peak have previously been implicated in the pathogenesis of cardiovascular disease. The base to base peak at chromosome 19 encompassed *FCAR* and *TNNT1* genes associated with coronary heart disease (Iakoubova et al., 2006; Guay et al., 2016) and *OSCAR* and *FPR2* genes associated with atherosclerosis plaque phenotype (Goettsch et al., 2011; Petri et al., 2015), whereas the base to base peak at chromosome 20 encompassed *ADAM33* and *TRIB3* associated with extent and promotion of atherosclerosis (Holloway et al., 2010; Formoso et al., 2011; Wang et al., 2012; Figarska et al., 2013; Prudente et al., 2015) and *HSPA12B* which is found to be enriched in atherosclerotic lesions (Han et al., 2003).

Our study presents the linkage analysis using extreme phenotype approach that was designed to capture region with genetic variants that have large effects on cIMT. Combination of linkage analysis in a large family-based study and exome sequence data provide a unique opportunity to explore the variants in the linkage regions. However, despite these distinct advantages, we were able to identify a genetic variant for only one of the several linked genomic regions, for which, there may be several reasons including structural variants, and intronic or intergenic single-nucleotide variants that were not evaluated in the current study. Interestingly, the 19q13.43, 20p13, and 21q22.12 linkage peaks were previously associated with various phenotypes in our study population including personality traits and depressive symptoms (Amin et al., 2012, 2017b).

## Conclusion

Our linkage analysis identified four genomic regions at 2p16.3, 19q13.43, 20p13, and 21q22.12 for cIMT. The significant linkage regions contain several plausible candidate genes. Further analyses are needed to demonstrate their involvement in atherosclerosis.

## Data Availability

The raw datasets are available on request.

## Author Contributions

DV, CvD, and NA contributed to the conceptualization and design of this work and were involved in interpretation of the results. DV and MK were involved in the analysis of the data. DV and NA were involved in writing and revising the manuscript. MG, RB, JvR, MvdH, RK, WvI, AU, and CvD were involved in data collection/preparation. MK, MG, RB, JvR, MvdH, RK, AU, WvI, and CvD contributed to the interpretation of the data and read and approved the final manuscript.

## Conflict of Interest Statement

The authors declare that the research was conducted in the absence of any commercial or financial relationships that could be construed as a potential conflict of interest.

## Footnotes

1 http://snp.gs.washington.edu/SeattleSeqAnnotation138/

2 http://evs.gs.washington.edu/EVS/

3 https://www.gtexportal.org/home/

4 https://www.qiagenbioinformatics.com/products/ingenuity-pathway-analysis/

5 http://www.genecards.org/cgi-bin/carddisp.pl?gene=PNPT1

## Abbreviations

BMI: body mass index
CADD: Combined Annotation-Dependent Depletion
cIMT: carotid intima-media thickness
ERF: Erasmus Rucphen Family
HLOD: heterogeneity logarithm of the odds
HWE: Hardy–Weinberg equilibrium
1kG: 1000 Genome Project
LOD: logarithm of the odds
MAF: minor allele frequency
NPL: non-parametric linkage
PCOS: polycystic ovary syndrome
POAG: primary open-angle glaucoma
SNV: single nucleotide variant
WHR: waist–hip ratio
