# Supplementary material for: Whole-Genome Linkage Scan Combined With Exome Sequencing Identifies Novel Candidate Genes for Carotid Intima-Media Thickness
Source: Front Genet. 2018 Oct 9;9:420. doi: 10.3389/fgene.2018.00420 (PMC6189289; doi:10.3389/fgene.2018.00420)
Supplement: Supplementary file 1 [file Table_1.docx]

***Supplementary Material***

**Whole-genome linkage scan combined with exome sequencing identifies novel candidate genes for carotid intima-media thickness**

**Dina Vojinovic, Maryam Kavousi, Mohsen Ghanbari, Rutger W.W. Brouwer, Jeroen G.J. van Rooij, Mirjam C.G.N. van den Hout, Robert Kraaij, Wilfred F.J. van IJcken, Andre G. Uitterlinden, Cornelia M. van Duijn, Najaf Amin***

***Correspondence:** Dr. Najaf Amin: n.amin@erasmusmc.nl

**Supplementary Methods**

**Replication effort**

The replication cohort included participants from the Rotterdam Study. The Rotterdam Study is a prospective, population-based cohorts study among the individuals living in the well-defined Ommoord district in the city of Rotterdam in the Netherlands (Ikram et al., 2017). The cohort was initially defined in 1990 among 7,983 persons, aged 55 years and older, who underwent a home interview and extensive physical examination at the baseline and during follow-up visits every 3-4 years (RS-I) (Ikram et al., 2017). The cohort was extended in 2000/2001 (RS-II, 3,011 individuals aged 55 years and older) and 2006/2008 (RS-III, 3,932 subjects, aged 45 and older). Written informed consent was obtained from all participants and the Medical Ethics Committee of the Erasmus Medical Center and the review board of The Netherlands Ministry of Health, Welfare and Sports approved the study. cIMT was measured using high-resolution B-mode ultrasonography with a 7.5-MHz linear array transducer (ATL UltraMark IV). Maximum cIMT was measured on longitudinal, two-dimensional ultrasound images of the near and far wall from both left and right arteries, as described previously (Bis et al., 2011). Genetic data of Rotterdam Study consisted of randomly selected individuals from the RS-I whose exomes were sequenced at an average depth of 54× (Nimblegen SeqCap EZ V2 capture kit) on an Illumina Hiseq2000 sequencer using the TruSeq Version 3 protocol (Amin et al., 2017; van Rooij et al., 2017). All Rotterdam Study samples were sequenced at the Human Genotyping Facility (HuGe-F) at the Department of Internal Medicine at Erasmus Medical Center, Rotterdam. The final dataset after quality control consisted of 600,806 SNVs in 2,356 individuals, of which 921 had cIMT data available. The variants identified under the linkage peaks using the exome sequencing data in the Erasmus Rucphen Family study were evaluated in the Rotterdam Study through the single variant association analyses of cIMT with the same adjustment as in discovery cohort using the RVtests software (Zhan et al., 2016).

**Supplementary Figures**

**(A)**


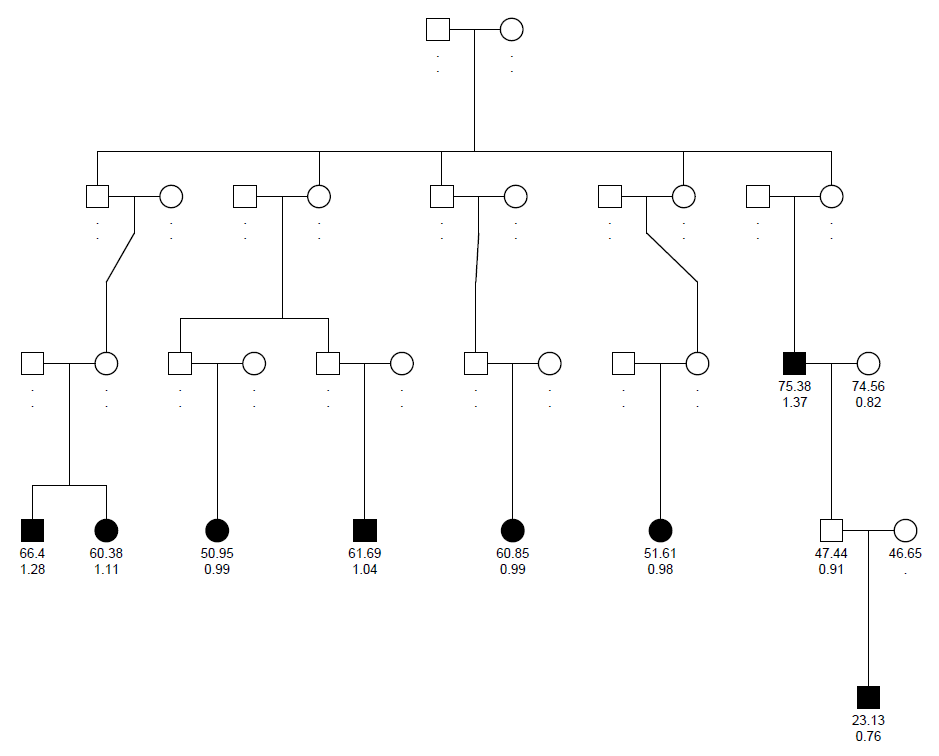


**(B)**


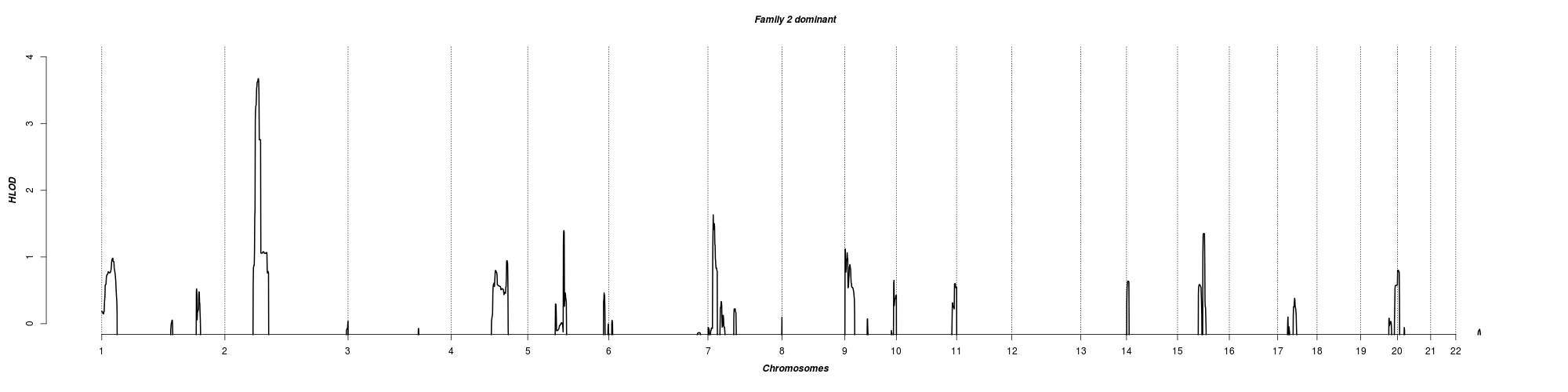


**Supplementary Figure 1.** The pedigree of highest HLOD score contributing family and the results of parametric per-family linkage analysis under the dominant model for the chromosome 2p16.3. (A) Squares represent males and circles females. Solid symbols depict affected family members. These family members were used in the linkage analysis. Age of individual and carotid intima-media thickness are displayed on the pedigree. Open symbols denote unaffected individuals (carotid intima-media thickness is displayed underneath the symbol) or individuals with no data available (carotid intima-media thickness is missing). (B) The x-axis shows 22 autosomal chromosomes, and the y-axis shows the heterogeneity LOD (HLOD) scores for the dominant model.

**(A)**


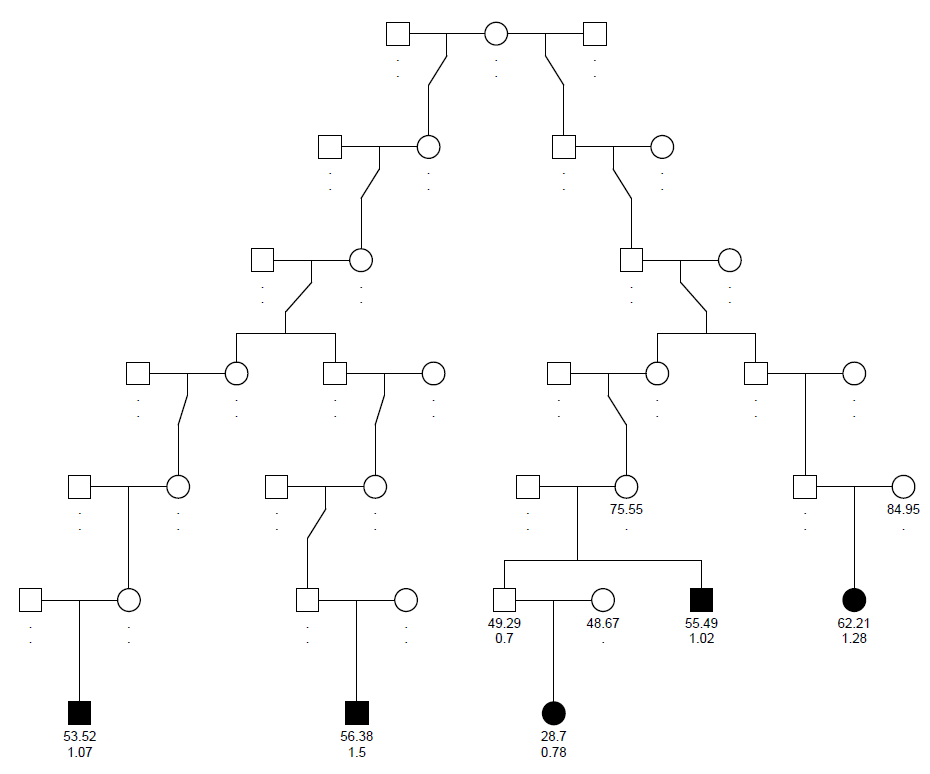


**(B)**


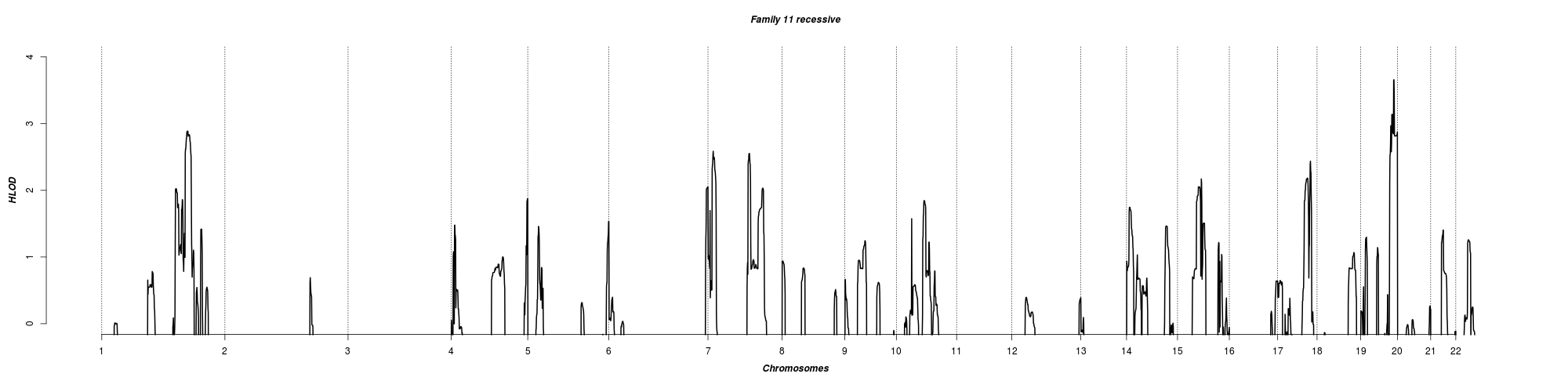


**Supplementary Figure 2.** The pedigree of highest HLOD score contributing family and the results of parametric per-family linkage analysis under the recessive model for the chromosome 19q13.43. (A) Squares represent males and circles females. Solid symbols depict affected family members. These family members were used in the linkage analysis. Age of individual and carotid intima-media thickness are displayed on the pedigree. Open symbols denote unaffected individuals (carotid intima-media thickness is displayed underneath the symbol) or individuals with no data available (carotid intima-media thickness is missing). (B) The x-axis shows 22 autosomal chromosomes, and the y-axis shows the heterogeneity LOD (HLOD) scores for recessive model.

**(A)**


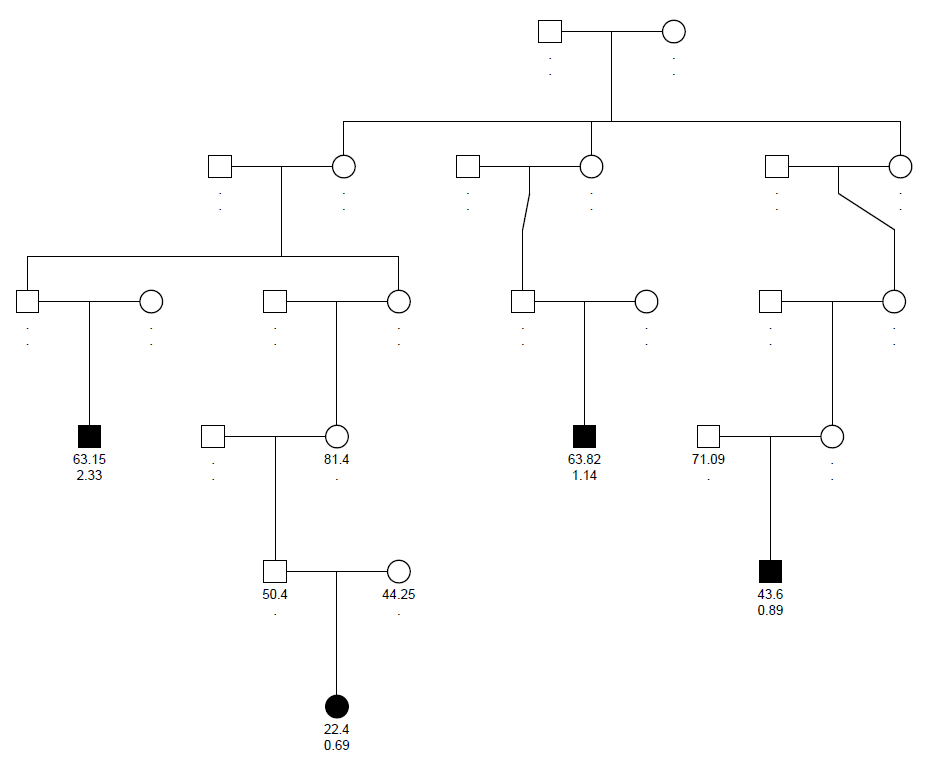


**(B)**

**
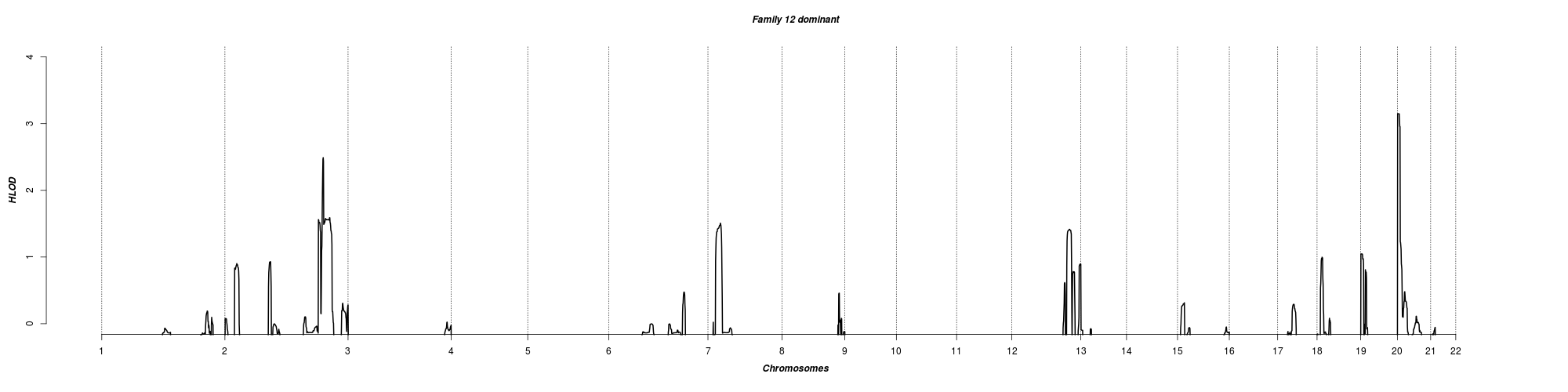
**

**Supplementary Figure 3.** The pedigree of highest HLOD score contributing family and the results of parametric per-family linkage analysis under the dominant model for the chromosome 20p13. (A) Squares represent males and circles females. Solid symbols depict affected family members. These family members were used in the linkage analysis. Age of individual and carotid intima-media thickness are displayed on the pedigree. Open symbols denote unaffected individuals (carotid intima-media thickness is displayed underneath the symbol) or individuals with no data available (carotid intima-media thickness is missing). (B) The x-axis shows 22 autosomal chromosomes, and the y-axis shows the heterogeneity LOD (HLOD) scores for the dominant model.

**(A)**


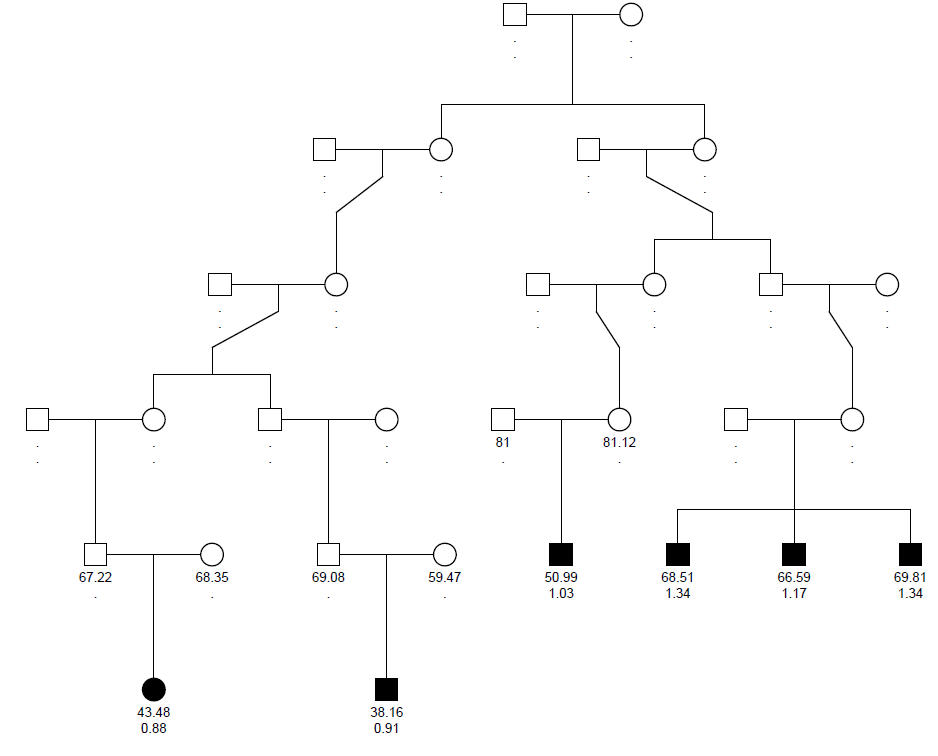


**(B)**

**
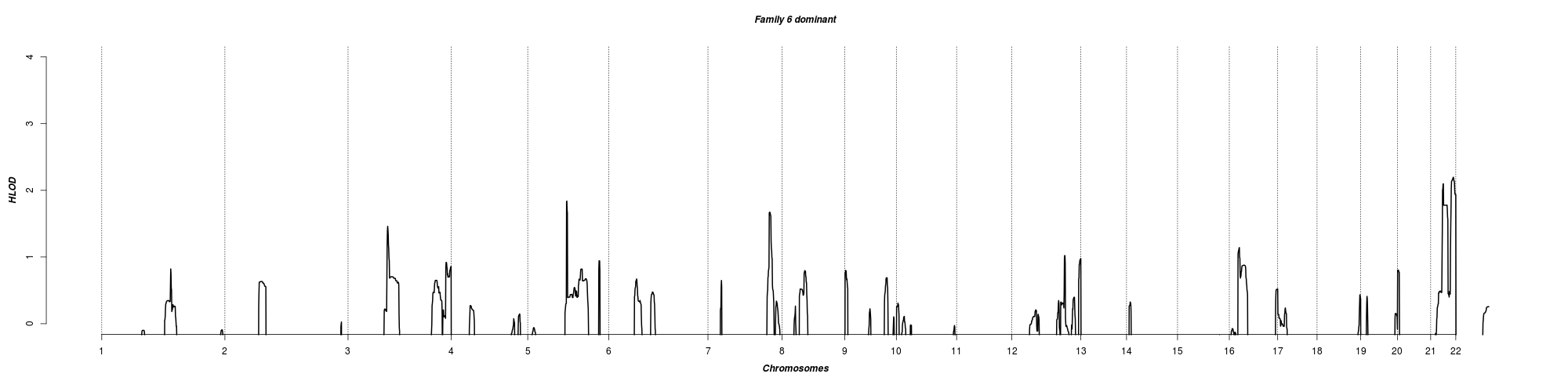
**

**Supplementary Figure 4.** The pedigree of highest HLOD score contributing family and the results of parametric per-family linkage analysis under the dominant model for the chromosome 21q22.12. (A) Squares represent males and circles females. Solid symbols depict affected family members. These family members were used in the linkage analysis. Age of individual and carotid intima-media thickness are displayed on the pedigree. Open symbols denote unaffected individuals (carotid intima-media thickness is displayed underneath the symbol) or individuals with no data available (carotid intima-media thickness is missing). (B) The x-axis shows 22 autosomal chromosomes, and the y-axis shows the heterogeneity LOD (HLOD) scores for the dominant model.

**
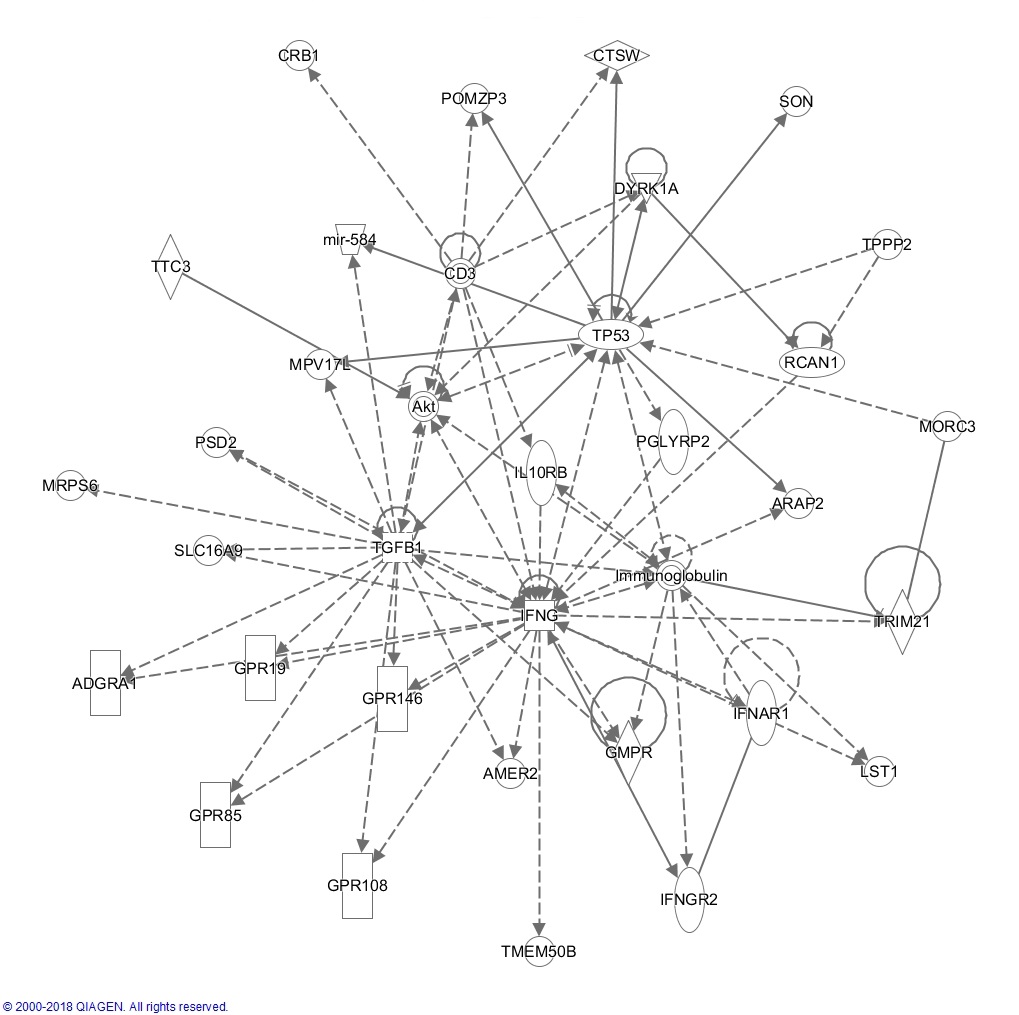
**

**Supplementary Figure 5.** Ingenuity Pathway Analysis tool network of genes under the linkage peak at 21q22 which are highly expressed in tissues relevant for atherosclerosis. Lines without arrow indicate interactions (chemical-chemical, protein-protein, chemical-protein, RNA-RNA, correlation) while lines with an arrow indicate activation, causation, expression, localization, transcription, molecular cleavage, membership, modification, phosphorylation, protein-DNA and/or protein-RNA interactions. Solid lines indicate direct interaction while dashed lines indicate indirect interactions. Diamond molecule shape denotes enzyme, rhombus - peptidase, inverted triangle - kinase, inverted trapezium - microRNA, circle in a circle - complex/group, vertical rectangle - G-protein coupled receptor, horizontal rectangle - ligand-dependent nuclear receptor, vertical ellipse - transmembrane receptor, horizontal ellipse - transcription regulator, square - cytokine, and circle - other.


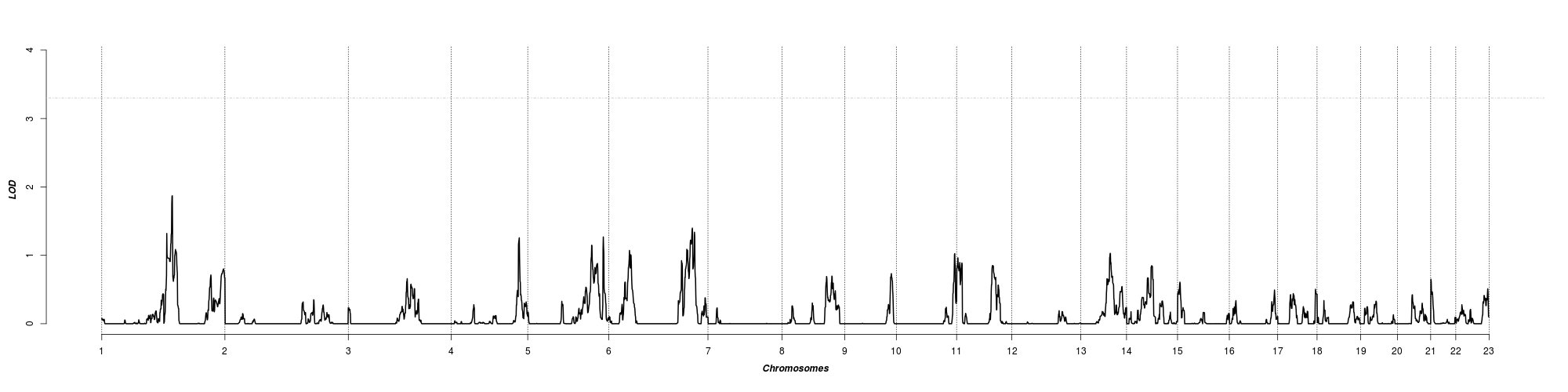


**Supplementary Figure 6.** The results of quantitative trait linkage analysis using variance component linkage.

**References**

Amin, N., Belonogova, N.M., Jovanova, O., Brouwer, R.W., van Rooij, J.G., van den Hout, M.C., et al. (2017). Nonsynonymous Variation in NKPD1 Increases Depressive Symptoms in European Populations. *Biol Psychiatry* 81(8)**,** 702-707. doi: S0006-3223(16)32669-5 [pii] 10.1016/j.biopsych.2016.08.008.

Bis, J.C., Kavousi, M., Franceschini, N., Isaacs, A., Abecasis, G.R., Schminke, U., et al. (2011). Meta-analysis of genome-wide association studies from the CHARGE consortium identifies common variants associated with carotid intima media thickness and plaque. *Nat Genet* 43(10)**,** 940-947. doi: ng.920 [pii] 10.1038/ng.920.

Ikram, M.A., Brusselle, G.G.O., Murad, S.D., van Duijn, C.M., Franco, O.H., Goedegebure, A., et al. (2017). The Rotterdam Study: 2018 update on objectives, design and main results. *European Journal of Epidemiology* 32(9)**,** 807-850. doi: 10.1007/s10654-017-0321-4.

van Rooij, J.G.J., Jhamai, M., Arp, P.P., Nouwens, S.C.A., Verkerk, M., Hofman, A., et al. (2017). Population-specific genetic variation in large sequencing data sets: why more data is still better. *Eur J Hum Genet* 25(10)**,** 1173-1175.

Zhan, X.W., Hu, Y.N., Li, B.S., Abecasis, G.R., and Liu, D.J.J. (2016). RVTESTS: an efficient and comprehensive tool for rare variant association analysis using sequence data. *Bioinformatics* 32(9)**,** 1423-1426. doi: 10.1093/bioinformatics/btw079.
